# Effects of dark septate endophytic fungi on the performance of non-mycorrhizal cabbage plants under normal and low water conditions

**DOI:** 10.3389/fmicb.2025.1593265

**Published:** 2025-07-08

**Authors:** Alena F. Lukács, Gábor Herczeg, Gábor M. Kovács

**Affiliations:** ^1^Department of Plant Anatomy, Institute of Biology, Eötvös Loránd University, Budapest, Hungary; ^2^HUN-REN-ELTE-MTM Integrative Ecology Research Group, Budapest, Hungary; ^3^Department of Systematic Zoology and Ecology, Institute of Biology, Eötvös Loránd University, Budapest, Hungary

**Keywords:** fungal inoculation, drought stress, endophytic fungi, organic farming, soil moisture, *Brassica oleracea*

## Abstract

Drought, a major consequence of global environmental change, poses a serious threat to both natural and agricultural ecosystems. Root-associated fungi, particularly the widely distributed dark septate endophytes (DSE), are key components of the plant microbiome and can influence host plant performance in various ways. We conducted two manipulative experiments using two model DSE species from a semiarid habitat to investigate their effects on a non-mycorrhizal host plant (cabbage) under both normal and reduced water supply conditions. The positive effects of *Periconia* were limited—it not only increased root biomass but also reduced water potential and soil moisture under normal watering conditions. In contrast, *Cadophora* significantly increased shoot biomass (by up to 50%) and root biomass in one experiment. However, this was also associated with a decline in plant water potential, particularly at the cost of reduced plant water status, and their effects varied on the same host. Interestingly, autoclaved inoculum, also had positive effects on plant growth. Our findings highlight the potential role of symbiotic DSE fungi in mitigating drought stress and suggest their promise as biotechnological tools for addressing the increasing challenges posed by drought.

## 1 Introduction

Climate change has multiple effects, with drought being a key factor that challenges natural biodiversity, agriculture, the economy, and society (Haile et al., 2020). Water deficit can affect plants in many ways, and drought tolerance can have morphological, physiological, and molecular biological backgrounds in plants (Basu et al., 2016; Gupta et al., 2020). Water availability is particularly crucial for crops such as cabbage, which have a high leaf area index, which leads to a relatively high transpiration rate and fast soil drying (Seidel et al., 2017). Given this trait, an adequate water supply is essential for cabbage, emphasizing the importance of appropriate irrigation management. The annual water demand of cabbage plants ranges from 380 to 500 mm, depending on the climate and the length of the growing season, with irrigation frequency varying between every 3 and 12 days (Food and Agriculture Organization-a, n.d.). Cabbage is an excellent source of vitamins (C, K, and A), minerals, and dietary fiber (Kumar et al., 2020). Over the past 15 years, global cabbage production has steadily increased, even though the area under cultivation has remained relatively stable, indicating an increase in production intensity (Food and Agriculture Organization-b, n.d.).

Plant-associated microbes are known to have a beneficial effect on the water status of the plants and can significantly enhance drought tolerance (Farooq et al., 2009; Ullah et al., 2019; Van Der Heijden et al., 1998). Microbiome-based and holobiont-centered approaches are increasingly recognized as important components of sustainable agriculture and green biotechnological solutions (Compant et al., 2019; Mitter et al., 2019; Trivedi et al., 2021). As a part of their microbiome, the majority of the terrestrial plants are colonized by different root-associated fungi (RAF), including mutualistic mycorrhizal fungi or root-colonizing endophytic fungi, the latter of which are less well understood (Porras-Alfaro and Bayman, 2011). Due to their positive effects, several commercial fungal products are used in agricultural practice; for example, arbuscular mycorrhizal fungal (AMF) inocula containing widely distributed AMF species of a wide host range are used to increase crop production and improve tolerance to abiotic and biotic stress factors (de Santana et al., 2014). Another frequent RAF group is the class 4 endophytes (Rodriguez et al., 2009), the Dark Septate Endophytes (DSEs), which are widespread root colonizer fungi capable of associating with a wide range of hosts, including non-mycorrhizal plants (Jumpponen and Trappe, 1998; Mandyam and Jumpponen, 2005). DSE fungi are a common, diverse group belonging to several orders of the phylum Ascomycota, including Helotiales, Xylariales, and Pleosporales (Jumpponen and Trappe, 1998; Knapp et al., 2012; Newsham, 2011). These fungi are typically distinguished by melanized septate hyphae and microsclerotia within the plant root cells (Jumpponen and Trappe, 1998). Despite their global presence and wide host range, their ecological function, physiology, and plant-fungal interactions remain poorly understood (Mayerhofer et al., 2013; Newsham, 2011).

The effect of DSE colonization on plant status can be negative, positive, or neutral (Jumpponen, 2001; Mandyam and Jumpponen, 2005; Newsham, 2011). Reininger and Sieber (2012) demonstrated the negative effect of certain DSE strains on the biomass production of spruce (*Picea* sp.). Tellenbach et al. (2011) found a neutral or negative effect of the DSE inoculation on Norway spruce (*Picea abies*). Mahmoud and Narisawa (2013) showed the positive effect of DSE (*Scolecobasidium humicola*) inoculation on tomato (*Solanum lycopersicum*) *in vitro* when an organic nitrogen source was applied. In a greenhouse, Vergara et al. (2017) gained similar results. They tested the effect of DSE on tomatoes under different N sources. DSE inoculation had a positive effect on the aboveground biomass production of tomato under an organic N source (Vergara et al., 2017). Andrade-Linares et al. (2011) also found that the DSE inoculation had a positive effect on tomato shoot biomass production, fruit yield, and quality in young plants. The DSE inoculation of Chinese wolfberry (*Lycium barbarum*) showed similar results. The 5-week-old plants produced enhanced total biomass and chlorophyll values in a potting experiment (Zhang et al., 2012). Khastini et al. (2012) showed in an *in vitro* experiment the reduction in Fusarium wilt (*Fusarium oxysporum*) disease and good growth of Chinese cabbage (*Brassica rapa* subsp. *pekinensis*) inoculated with DSE (*Veronaeopsis simplex*). The control of Fusarium wilt disease in melon (*Cucumis melo*) by DSE (*Cadophora* sp.) was demonstrated in a potting experiment (Khastini et al., 2014). Newsham (2011) demonstrated the commonly positive effect of DSE inoculation in a meta-analysis of controlled experimental studies. Based on these studies demonstrating the positive effect on plant growth and increased resistance against pathogens, DSE fungi can be used in agriculture and horticulture as inocula to improve the production quantity and pathogen or drought tolerance, likewise, mycorrhizal fungi.

DSE fungi are commonly present in abiotic-stressed areas like arid or semiarid ecosystems (Knapp et al., 2012; Mandyam et al., 2012). Thus, DSE colonization is likely crucial for host plants in these harsh environments. Two characteristic and common DSEs were found colonizing grass and non-grass hosts in semiarid sandy grasslands, *Periconia macrospinosa* and *Cadophora,* considered generalist root colonizers (Knapp et al., 2012). The complete genome of these two DSEs has been sequenced (Knapp et al., 2018), and these fungi have been used in different experiments, among them with horticultural crops (Yakti et al., 2018, 2019). DSE symbiosis could be particularly important for non-mycorrhizal crops, such as cabbage (*Brassica* sp.), where AMF inoculation cannot be applied. The *Brassicaceae* family encompasses several vegetable crops of nutritional importance, including cauliflower, broccoli, kale, and cabbage. The important model plant *Arabidopsis thaliana* also belongs to this family, with many experiments carried out in connection with DSE inoculation (Mandyam and Jumpponen, 2013, 2015).

The positive effects of DSE fungi on biotic and abiotic stress tolerance of plants and plant growth have been widely demonstrated. This raised the question of whether DSE fungi could be developed into beneficial inocula for mycorrhizal fungal preparations in agriculture and horticulture. In this study, we aimed to test the hypothesis that the individual inoculation with two different DSE fungi, *Cadophora* sp. and *Periconia macrospinosa* strains originating from a semi-arid habitat, and the coinoculation with these two, would affect a non-mycorrhizal plant under both normal and reduced water supply. We used cabbage (*Brassica oleracea* var. *capitata*), an important non-mycorrhizal vegetable crop with high water demand; thus, any positive effect on its water status might have significant practical importance.

## 2 Materials and methods

### 2.1 DSE inoculum preparation

In our previous study of root endophytes, several DSE fungi were isolated and identified from a semiarid grassland area (Knapp et al., 2012; Knapp et al., 2015). Among them, a collection of *Cadophora* sp. representatives (see Knapp et al., 2012) was frequently isolated from non-grass hosts in our sampling areas. Based on a pilot experiment with six different isolates [REF001, REF013, REF018, REF024, REF036, and REF044 in Knapp et al. (2012)], a *Cadophora* strain [Cado018 – REF018 in Knapp et al. (2012)] was chosen based on its growth in liquid medium and colonization of cabbage. The pilot experiment was conducted similarly to Experiment I for 8 weeks, with the roots exclusively screened microscopically (see below). As *P. macrospinosa* generally colonized the roots of grass species, we used the DSE2036 strain, the isolate with a fully sequenced genome (Knapp et al., 2018) and with previous experimental studies (Yakti et al., 2019). The selected strains were grown on modified Melin-Norkrans medium (MMN) (Marx, 1969) plates covered with cellophane for 6 days. Afterward, the mycelia of DSE strains were cut into small pieces and placed into a 1.5 mL Eppendorf tube containing glucose-free MMN liquid medium and then vortexed three times for 10 min. These stock inocula were placed into 15 mL Falcon tubes filled with glucose-free MMN liquid medium and grown for 1 week with daily vortexing (for 5 min each time). After 7 days, two 15 mL inocula were transferred into 1 L of glucose-free MMN liquid medium and grown for 10 days with daily shaking.

### 2.2 Experiment I

Cabbage (*Brassica oleracea* var. *capitata*, “Zeusz F1” – from ZKI Research Institute of Vegetable Production Ltd., Kecskemét, Hungary) seeds were placed into a plant tray filled with peat substrate (Pindstrup Plus Blue, Pindstrup); both seeds and the peat were commercial products and were handled in our laboratory with care, so we essentially considered them free of root-colonizing and/or pathogenic fungi. Two-week-old seedlings were planted into 450 mL plastic pots (one seedling per pot) filled with a mixture of twice-autoclaved sand and zeolite (2:1). In this experiment, DSE inoculum was prepared from the *Cadophora* strain. We employed two treatments: inoculation with *Cadophora* strain *vs.* autoclaved *Cadophora* strain (considered mock control) and normal *vs.* reduced water levels. Half (*N* = 18) of the cabbage seedlings were inoculated with 25 mL of *Cadophora* inoculum, and 75 mL of sterilized tap water was added. The other half (*N* = 18) of cabbage seedlings were inoculated with 25 mL of autoclaved (twice) *Cadophora* inoculum and also watered with 75 mL of sterilized tap water. We randomly separated the seedlings into two water treatment groups (*N* = 9 each) within the inoculation treatments. Plants were irrigated every 3 days with sterilized tap water. In the reduced water treatment, seedlings received 40 mL of tap water per irrigation, while in the normal treatment, seedlings received 80 mL per irrigation, which was determined in the pilot experiments to be the optimal water supply for cabbage in our experimental conditions. The pots were randomly rearranged three times a week. The plants were grown for 20 weeks at room temperature (21–24°C) in a plant-growing room with natural sunlight, after which they were harvested.

### 2.3 Experiment II

Similarly to Experiment I, cabbage (*B. oleracea* var. *capitata*, “Zeusz F1” – from “Zöldségtermesztési Kutató Intézet” (ZKI), Kecskemét, Hungary) seeds were placed into a plant tray filled with peat substrate (Pindstrup Plus Blue, Pindstrup). The 2-week-old seedlings were planted into 450-mL plastic pots (one seedling per pot) filled with a mixture of twice autoclaved sand and zeolite (2:1). In this experiment, we used DSE inocula from both *Cadophora* and *P. macrospinosa* strains. We employed two treatments: one was the inoculation, where we used *Cadophora* (C)*, P. macrospinosa* (P), and *Cadophora* + *P. macrospinosa* (C + P), and autoclaved *Cadophora* + *P. macrospinosa* (aut. C + P) and the control (Control); the other treatment was the water with normal *vs.* reduced levels. We randomly selected 24 cabbage plants for the inoculation treatment for each of the five treatment groups. The first treatment group was inoculated with 25 mL of *Cadophora* inoculum, the second with 25 mL of *P. macrospinosa* inoculum, the third with 15–15 mL of *Cadophora* and *P. macrospinosa* inocula, and the fourth with 15–15 mL of autoclaved *Cadophora* and *P. macrospinosa* inocula, all being watered with 75 mL of sterilized tap water. The fifth treatment group was treated with 100 mL of sterilized tap water. We randomly separated the seedlings into two water treatment groups (N = 12 each) within the inoculation treatments. Plants were irrigated every 3 days with sterilized tap water. Seedlings in the reduced water treatment received 40 mL, while those in the normal water treatment received 80 mL of tap water/irrigation (see Section 2.2). The plants were grown for 12 weeks at room temperature under natural light conditions. Due to the time of year when the experiment was conducted, additional artificial light with a plant growth-promoting LED was also applied. The pots were randomly rearranged every 3 weeks, and the plants were harvested after 12 weeks.

### 2.4 Measurements

In both experiments, the measurements were conducted following the same protocol. When harvesting the plants, the soil moisture content was measured with a soil moisture meter (Basetech BT-235PT, China). The leaf water potential was measured with the “Schulander” type pressure chamber (ARIMAD 3000, MRClab, Holon, Israel) using nitrogen gas immediately after harvesting. To check the presence of DSEs in pots inoculated with living fungi and to test the lack of DSEs in controls, randomly chosen segments (~100 cm in total) from the separated root system were stained using the ink-vinegar method (Vierheilig et al., 1998). The roots were kept at 90°C for 20 min in 10% KOH and washed three times with distilled water. The washed roots were treated with 5% acetic acid (Reanal, Hungary). Roots were stained in a 5% blue ink (Schaeffer, USA) acetic acid solution at 90°C for 8 min. The stained roots were washed in 5% acetic acid and then mounted in 90% lactic acid (Reanal, Hungary). The DSE colonization was checked with a light microscope (Nikon, Eclipse, E200, Japan). Based on our experiences, correcting the total root biomass by removing the root for DSE colonization checking was unnecessary, as removing this amount of root had no significant effect on the total root mass. Shoots and roots were separated and dried at 65°C for approximately 2 weeks until constant weight was achieved. Subsequently, the dry biomass of shoots and roots was measured.

### 2.5 Statistical analyses

In both experiments we had four variables describing the relevant characteristics of the host plant and its environment: “shoot dry mass” (hereafter: shoot), “root dry mass” (hereafter: root), “plant water potential” (hereafter: water), and “soil moisture” (hereafter: soil). As these variables were not independent, we decided to first run multivariate linear models (mLMs), taking the non-independence into account. We chose *Pillai’s Trace* as a test statistic because it is generally considered robust with respect to model assumptions. After detecting significant effects in the multivariate linear models (mLMs), we run univariate linear models (LMs) for each individual trait (shoot dry mass, root dry mass, plant water potential, and soil moisture) to assess the effects of inoculation and water treatments.

Models were built similarly for both experiments. Shoot, root, water potential, and soil moisture were included as response variables in the mLMs, while water and inoculation treatments, along with their interaction, were entered as fixed explanatory effects. In both mLMs, all single and interaction explanatory terms were significant (see 3.); therefore, we proceeded to run subsequent LMs for each response variable using the same fixed effect structure. Additionally, we aimed to assess the direct effects of our treatments on the shoot-to-root ratio. This variable could not be included in the mLMs, as it is derived from two response variables already included in the model. As a result, we conducted separate LMs for the shoot-to-root ratio, following the same approach as for the other variables.

Model residuals were checked via Q-Q plots. Groups were compared by inspecting the presence or absence of overlap between 85% confidence intervals (CIs). It has been shown that the absence of overlap between ca. 83–84% CIs is equivalent to *p* < 0.05 (Payton et al., 2003); hence, 85% CIs can be used as reliable indicators of statistical difference (Horváth et al., 2023). Linear models were run in R 4.2.2 (R Core Team, 2022), and estimated marginal means and their confidence intervals (CIs) were extracted using the *emmeans* package (Lenth, 2019).

## 3 Results

### 3.1 Experiment I

There were no visible disease symptoms on the roots and shoots of the cabbage plants during the 20-week-long experiment. Characteristic structures for DSE colonization (melanized and hyaline septate hyphae, microsclerotia) were found in the roots. Moreover, extraradical hyphal structures could be detected in the roots of all plants inoculated with live *Cadophora*. No DSE colonization was detected in the roots of the plants inoculated with autoclaved *Cadophora*.

**Figure 1:**
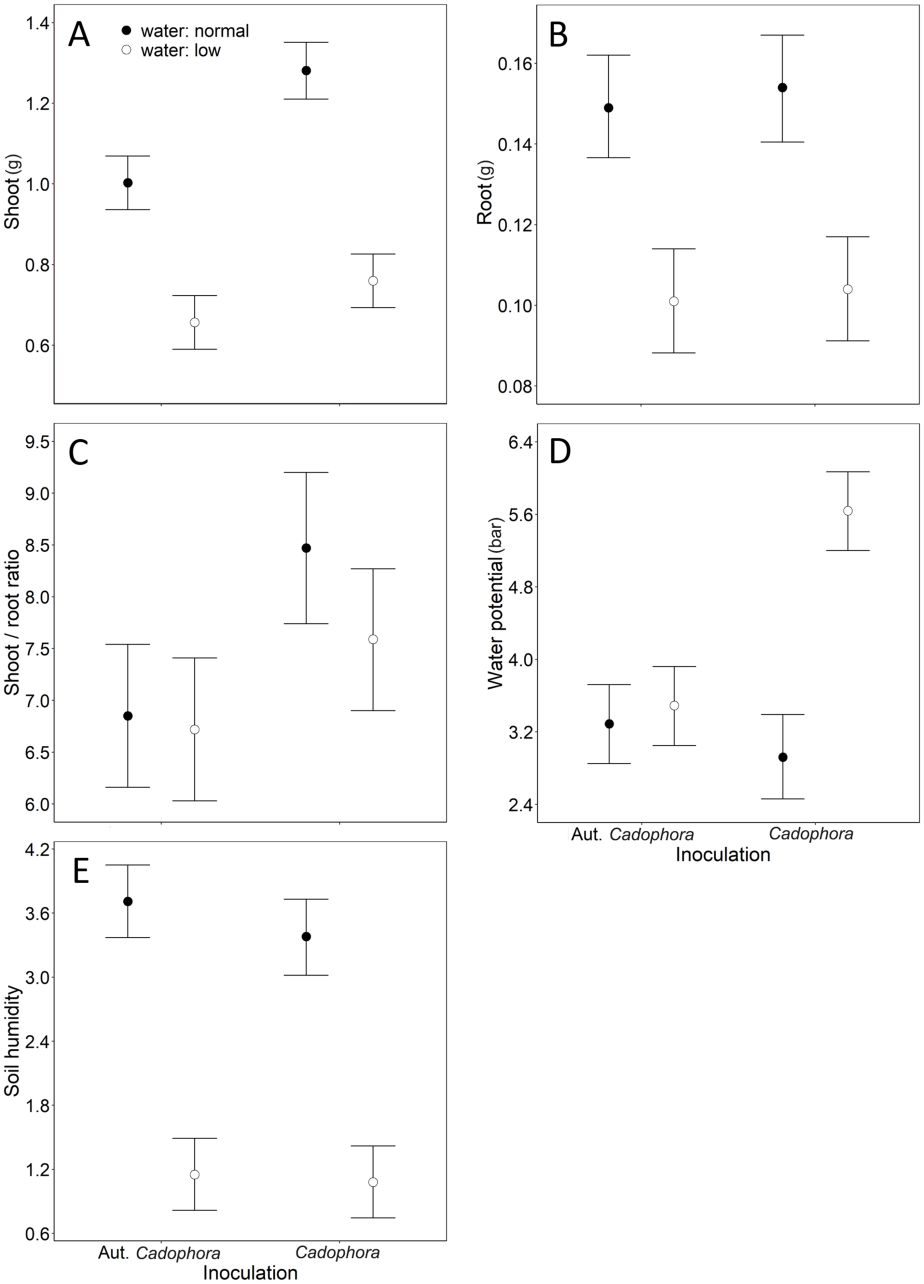
The combined effects of water and inoculation treatments from the 20-week-long Experiment I, where we studied the effect of two treatments (inoculation with *Cadophora* strain *vs.* autoclaved *Cadophora* strain (considered as mock control) and with normal *vs.* reduced levels of water) on cabbage plants. Means + 85% Confidence Intervals are shown, following Payton et al. (2003), to provide a clear graphical comparison of the experimental groups. ‘Aut.’ denotes autoclaved. Significant effects (see Table 1 for numerical details): **(A)** water treatment and inoculation treatment on shoot dry weight; **(B)** water treatment on root dry weight; **(C)** inoculation treatment on the shoot-to-root ratio, **(D)** water treatment, inoculation treatment and water × inoculation treatment interaction on water potential; **(E)** water treatment on soil moisture. Note that for water potential **(D)**, higher values represent lower water potential.

All effects in our mLM were highly significant (water: *Pillai’s Trace*_1,31_ = 0.85, *p* < 0.001; inoculation: *Pillai’s Trace*_1,31_ = 0.52, p < 0.001; water × inoculation: *Pillai’s Trace*_1,31_ = 0.45, *p* = 0.002). Shoot biomass was significantly affected by both water treatment and inoculation, with a marginally significant interaction also detected (Table 1). Reduced watering led to a decrease in shoot mass, while inoculation with living *Cadophora* increased it (Figure 1A). Under normal watering conditions, *Cadophora* inoculation increased shoot biomass by more than 20% (1.00 ± 0.13 g vs. 1.23 ± 0.24 g) and by more than 10% (0.66 ± 0.11 g vs. 0.76 ± 0.08 g) under reduced watering (Figure 1A). The interaction suggests that the positive effect of living *Cadophora* is especially evident under normal watering conditions (Figure 1A). Root biomass was affected only by water treatment (Table 1), with reduced watering resulting in lower root mass (Figure 1B). The shoot-to-root ratio was affected solely by inoculation (Table 1), with living *Cadophora* increasing the ratio (Figure 1C). Water potential was significantly affected by water treatment, inoculation, and their interaction (Table 1). Both reduced watering and inoculation with living *Cadophora* led to lower water potential (Figure 1D). The interaction showed that the combination of reduced watering and living *Cadophora* resulted in an extremely low water potential, while the other treatment combinations showed similar values (Figure 1D). Soil moisture was affected only by water treatment (Table 1), with reduced watering leading to decreased soil moisture (Figure 1E).

**Table 1:** Results of the trait-by-trait linear models from the 20-week-long Experiment I, where we studied the effect of two treatments (inoculation with *Cadophora* strain vs. autoclaved *Cadophora* strain (considered as mock control) and with normal vs. reduced levels of water) on cabbage plants.

Effect	Shoot		Root		Shoot: root ratio		Water potential		Soil	
	*F*(df1,df2)	*P*	*F*(df1,df2)	*P*	*F*(df1,df2)	*P*	*F*(df1,df2)	*P*	*F*(df1,df2)	*P*
Water (W)	**87.05** (1,31)	**<0.001**	**31.55** (1,31)	**<0.001**	0.94 (1,31)	0.34	**23.47** (1,31)	**<0.001**	**110.02** (1,31)	**<0.001**
Inoculation (I)	**16.96** (1,31)	**<0.001**	0.19 (1,31)	0.67	**6.75** (1,31)	**0.014**	**9.72** (1,31)	**0.004**	0.72 (1,31)	0.40
W × I	*3.69* (1,31)	*0.064*	0.01 (1,31)	0.92	0.62 (1,31)	0.43	**17.62** (1,31)	**<0.001**	0.32 (1,31)	0.57

Significant effects (*p* < 0.05) are in bold. Marginally significant effects (0.05 < *p* < 0.1) are italicized.

### 3.2 Experiment II

There were no visible disease symptoms in the roots and shoots of cabbage plants during the 12-week-long experiment. DSEs and their characteristic structures (melanized and hyaline septate hyphae, microsclerotia) were found in the roots. Moreover, extraradical hyphal structures could be detected in the roots of all plants of all treatment groups inoculated with living DSE. No DSE colonization was detected in the roots of the control plants, and the plants were inoculated with autoclaved DSE.

**Figure 2:**
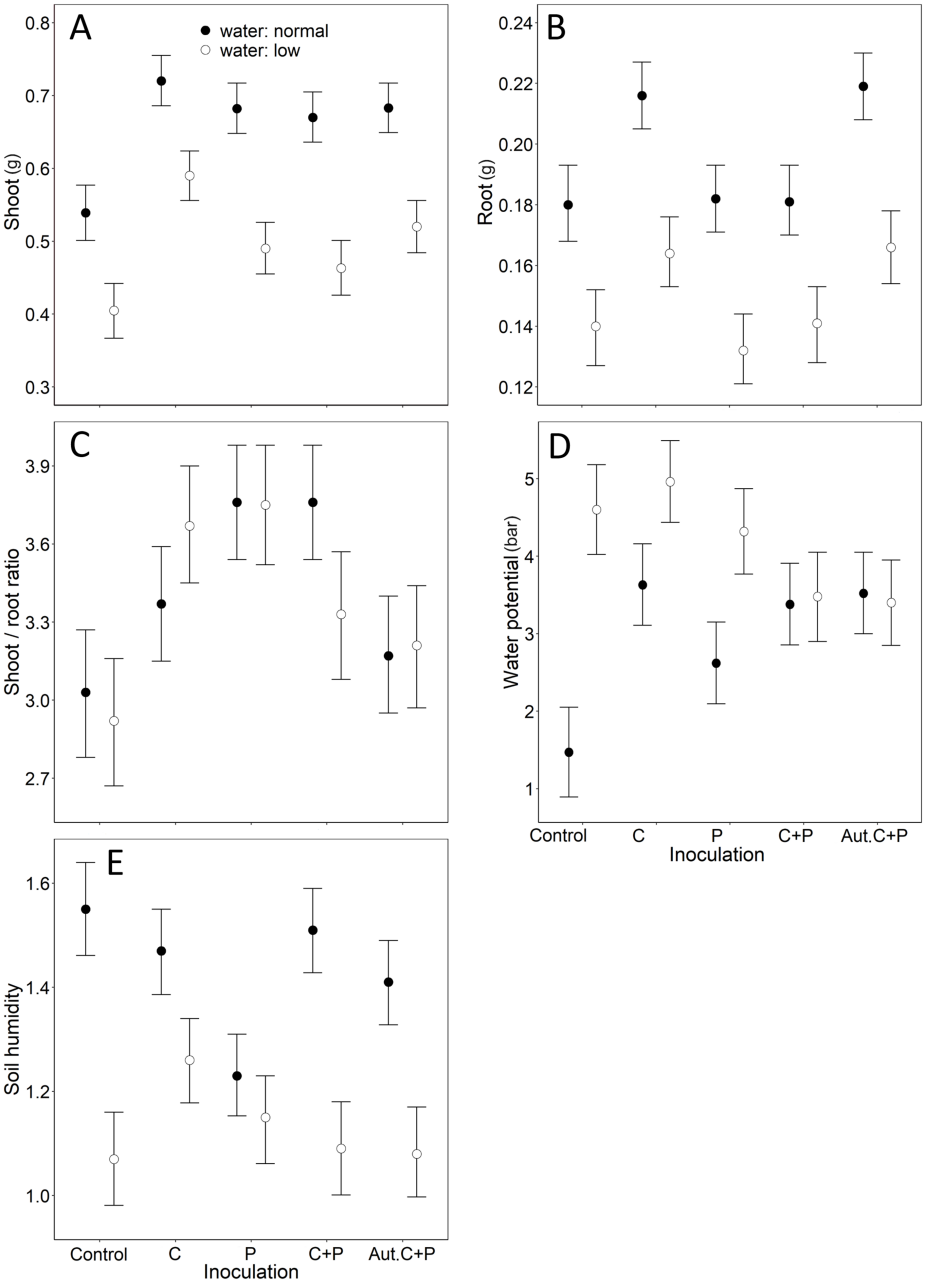
The combined effects of water and inoculation treatments in the 12-week-long Experiment II, where we studied the effect of two treatments (inoculation, where we used *Cadophora*, *Periconia macrospinosa*, *Cadophora* + *P. macrospinosa,* and autoclaved *Cadophora* + *P. macrospinosa,* and the control; the other treatment was water with normal vs. reduced levels) on cabbage plants. Means + 85% Confidence Intervals are shown, following Payton et al. (2003), to provide a clear graphical comparison of the experimental groups. ‘C’ denotes *Cadophora* sp., ‘P’ denotes *Periconia macrospinosa*, ‘Aut.’ denotes autoclaved. Significant effects (see Table 2 for numerical details): **(A)** water treatment and inoculation treatment on shoot dry weight; **(B)** water treatment and inoculation treatment on root dry weight; **(C)** inoculation treatment on the shoot-to-root ratio, **(D)** water treatment, inoculation treatment and water × inoculation treatment interaction on water potential; **(E)** water treatment, inoculation treatment and water × inoculation treatment interaction on soil moisture. Note that for water potential **(D)**, higher values represent lower water potential.

All effects in our mLM were highly significant (water: *Pillai’s Trace*_1,102_ = 0.75, *p* < 0.001; inoculation: *Pillai’s Trace*_4,102_ = 0.71, *p* < 0.001; water × inoculation: *Pillai’s Trace*_4,102_ = 0.38, *p* < 0.001). The shoot was affected by water treatment and inoculation (Table 2). Reduced watering decreased shoot biomass (Figure 2A). All inoculation types resulted in higher shoot biomass than the control, with *Cadophora* inoculation having a stronger effect than the rest (Figure 2A). Under normal watering, inoculation with living *Cadophora* increased shoot biomass by more than 30% compared to the control (0.54 ± 0.03 g vs. 0.72 ± 0.11 g) and by more than 50% under reduced watering (0.39 ± 0.06 g vs. 0.59 ± 0.05 g) (Figure 2A). Root biomass was also affected by both water treatment and inoculation (Table 2), with reduced watering resulting in decreased root biomass (Figure 2B). Inoculation with *Cadophora* and autoclaved DSE resulted in higher root biomass than the control, while inoculation with *Periconia* or *Cadophora* + *Periconia* did not (Figure 2B). The shoot-to-root ratio was only affected by inoculation (Table 2): all inoculations increased the ratio, but the autoclaved DSE (Figure 2C). Water potential was affected by water treatment, inoculation, and their interaction (Table 2). Reduced watering resulted in lower water potential (Figure 2D). *Cadophora* inoculation decreased water potential compared to the control, while the other inoculations did not (Figure 2D). However, the interaction revealed a more complex picture. In the reduced watering treatment, inoculation with autoclaved DSE increased water potential compared to the control, while the other inoculations did not (although the *Cadophora* + *Periconia* inoculation tended to Figure 2D). In the normal watering treatment, all inoculations decreased water potential compared to the control (Figure 2D). Soil was affected by water treatment, inoculation and their interaction (Table 2). Reduced watering resulted in lower soil moisture (Figure 2E). *Periconia* inoculation decreased soil moisture compared to the control, while *Cadophora* inoculation resulted in higher soil moisture than *Periconia* or autoclaved DSE inoculation (Figure 2E). Again, the interaction revealed a complex picture. In the reduced watering treatment, *Cadophora* inoculation resulted in higher soil moisture compared to the control, while the other inoculations did not (Figure 2E). Under normal watering, *Periconia* inoculation resulted in lower soil moisture than the other treatments (Figure 2E).

**Table 2:** Results of the trait-by-trait linear models from the 12-week-long Experiment II, where we studied the effect of two treatments (inoculation, where we used *Cadophora* and *Periconia. macrospinosa*, *Cadophora* + *P. macrospinosa,* and autoclaved *Cadophora* + *P. macrospinosa* and the control; the other treatment was the water with normal vs. reduced levels) on the cabbage plant.

Effect	Shoot		Root		Shoot-to-root ratio		Water potential		Soil	
	*F*(df1, df2)	*P*	*F*(df1, df2)	*P*	*F*(df1, df2)	*P*	*F*(df1, df2)	*P*	*F*(df1, df2)	*P*
Water (W)	**114.31** (1,102)	**< 0.001**	**86.68** (1,102)	**< 0.001**	0.16 (1,102)	0.68	**25.26** (1,102)	**< 0.001**	**65.76** (1,102)	**< 0.001**
Inoculation (I)	**14.34** (4,102)	**< 0.001**	**9.87** (4,102)	**< 0.001**	**7.55** (4,102)	**< 0.001**	**3.02** (4,102)	**0.02**	**2.88** (4,102)	**0.03**
W × I	0.96 (4,102)	0.43	0.27 (4,102)	0.90	1.42 (4,102)	0.23	**5.83** (4,102)	**< 0.001**	**3.70** (4,102)	**0.007**

Significant effects (*p* < 0.05) are in bold. Marginally significant effects (0.05 < *p* < 0.1) are italicized.

## 4 Discussion

In agriculture, especially under already dry conditions, massive investments into irrigation systems or other water management methods are needed (Zinkernagel et al., 2020). Successful anti-drought biological treatments that do not directly provide extra water are highly interesting. In this study, we investigated whether root-associated endophytic fungi originating from semiarid environments could enhance the growth performance of non-mycorrhizal vegetable crops under different water regimes. Our key findings are that DSE fungi were able to colonize cabbage and (i) enhanced both shoot and root growth, (ii) the two different DSE fungi had different effects, *Cadophora* being more beneficial for the plant than *P. macrospinosa*, (iii) the growth effects were typically positive but came at a cost of water status, corresponding with the increasing the shoot-to-root ratio, and (iv) autoclaved DSE also had positive effects, though with fewer costs.

Several studies have investigated the impact of DSE inoculation on plant development, biomass production, photosynthesis, and plant metabolism (Andrade-Linares et al., 2011; Mahmoud and Narisawa, 2013; Newsham, 2011; Yakti et al., 2018, 2019). Plant biomass is one of the most widely used indicators of plant growth. In our case, it was a key focus: shoot biomass is economically important in cabbage, while root biomass is important for plant performance, especially under water shortage. In our first experiment, live *Cadophora* performed better than autoclaved *Cadophora* in promoting shoot development. However, this result must be considered with caution because, as the second experiment also demonstrated in our system, autoclaved DSE could affect plant development *in itself,* and thus we had only the protocol-based control for comparisons in the first experiment. In the second experiment, all inoculation types led to increased shoot development compared to the control, with live *Cadophora* outperforming the rest significantly. The fact that living *Cadophora* outperformed the mixed living *Cadophora* + *P. macrospinosa* inoculation highlights the complexity and unpredictability of fungus–fungus interactions. Root development was enhanced by the presence of live *Cadophora* and the autoclaved DSE mix. These results suggest that both live and autoclaved DSE can improve cabbage growth. However, the effect is strongly dependent on the DSE species, positioning *Cadophora* as a promising candidate for cabbage cultivation. Of particular interest are the significant positive effects of autoclaved DSE on both shoot and root development. Although DSE colonization is not necessarily needed for a positive effect on inoculated plants (Newsham, 2011), our results warrant further investigation to (i) elucidate the mechanisms behind the comparable effects of living *vs.* autoclaved DSE and (ii) to explore the feasibility and potential costs and benefits of using autoclaved DSE as a biostimulant in agriculture.

Previous results suggested that *P. macrospinosa* inoculation was more effective with grasses than non-grass plants (Mandyam et al., 2012). In contrast, *Cadophora* DSE strains have been isolated primarily from non-grass plants (Knapp et al., 2012, 2018). This association with grass *vs.* non-grass hosts may explain why the effect was less pronounced for *P. macrospinosa* than for *Cadophora* in our cabbage experiments. Our results on species-specific DSE effects are congruent, e.g., with those achieved by Li et al. (2019), who reported that *Paraphoma* sp. and *Cladosporium* sp. strains enhanced the root weight of *Glycirrhiza uralensis*. However, *Embellisia* sp. and *Cladosporium* sp. strains had the most beneficial effects on *Zea mays*. Additionally, differences detected at different cabbage ages (12 *vs*. 20 weeks) highlight the effects that manifest variably during plant development. For instance, the positive *Cadophora* effect on root development detected in 12-week-old cabbage, but not in 20-week-old cabbage, shows that there might be an important effect in early developmental stages. In line with our results, Andrade-Linares et al. (2011) found that the shoot biomass of young tomato plants, 6 weeks after the inoculation, was affected positively by DSE inoculation, but these positive effects were not observed on 24-week-old tomato plants.

The specificity of the DSE effects on certain plant parts and/or organs is an important question. Nevertheless, previous results on the effects of DSE on the shoot-to-root ratio are variable. Newsham (2011) found, in a meta-analysis, that DSE generally did not influence the root-to-shoot ratio. According to Hou et al. (2021), the effect of DSE inoculation on the shoot-to-root ratio of *Artemisia ordosica* plants depends on environmental factors like soil moisture, nutrient content, pH, and plant and fungus species. They found that DSE could decrease the shoot-to-root ratio under increasing NaCl concentration. Both of our experiments revealed a significant effect of the inoculation on the shoot-to-root ratio. In Experiment I, living *Cadophora* increased the ratio compared to autoclaved *Cadophora*. In Experiment II, all inoculations increased the ratio compared to the control, but the autoclaved DSE mix showed the same trend; it was not significant. All inoculations with live DSE increased the ratio, while autoclaved DSE had a weak effect at best. These results are congruent with the results gained from analyzing shoot and root separately, showing that the main positive DSE effects are observable in shoot development, even in the case of *Cadophora*, which also increased root mass in 12-week-old cabbage. Experiment II suggests that the effect of autoclaved DSE is less plant-part specific than the effect of living DSE.

Some previous studies have also considered ecologically relevant interactions by examining DSE effects under various conditions. For instance, Yakti et al. (2019) examined the effect of *Cadophora* and *P. macrospinosa* strains, the same DSE fungi used here, on tomato growth in the presence of organic and inorganic N and P sources. When organic nutrient resources were present, only the *P. macrospinosa* strain increased both the shoot and root weight of tomato plants, in contrast to the case when inorganic forms were provided, and both *Cadophora* and *P. macrospinosa* increased only shoot biomass. Mandyam and Jumpponen (2015) found that *P. macrospinosa* can promote growth in *A. thaliana* when organic nutrients are supplied, unlike untreated plants. Furthermore, a few pioneering studies have also investigated the effects of DSE under various water regimes. Some DSE strains isolated from wild rice (*Oryza glumaepatula*) could improve the growth of rice under drought stress (Santos et al., 2017). The drought resistance of sorghum could be positively affected by *Exophiala pisciphila* isolate with better plant growth parameters, gas exchange, photosynthesis, secondary metabolites, and enzyme activities (Zhang et al., 2017). Our study found that there were limited ecologically relevant interactions between inoculation and water treatments, affecting plant growth. In Experiment I, the positive effect of living *Cadophora* over autoclaved *Cadophora* on the shoot development of 20-week-old cabbage was mainly present under a normal water regime. However, the interaction was only marginally significant (*p* = 0.064). In Experiment II, we found no interaction effect on shoot or root development in 12-week-old cabbage. Our reduced water treatment had a strong overall effect on plant development by decreasing both shoot and root mass. It also decreased the plants’ water potential and soil moisture. Hence, the lack of interaction is not a result of an unsuccessful water treatment. In our system, the positive effects of DSE (living or autoclaved) on plant growth are present in both favorable and harsh water conditions.

Considering the various positive effects of DSE inoculation on plant growth, regardless of water treatment, it is important to ask whether these improvements came without associated costs. Potential costs regarding water usage (soil moisture) and water status (water potential) could be directly estimated in our experimental setup. Even though it is a fundamental question for both understanding fungus–plant symbiosis and for applying living or autoclaved DSE in agriculture for increasing yield, the effects of DSE inoculation on soil moisture remain poorly understood. This factor may also play a critical role in plant–plant competition, especially in natural ecosystems. The effect on plant water potential could be explained by the positive effect of DSE inoculation on the shoot-to-root ratio. The relative increase of the aboveground parts could lead to higher transpiration, while a concurrent decrease in root mass may limit water uptake. When a reduced water regime was applied in the *Cadophora* inoculation case, the soil’s water content was higher than in the control, suggesting complex interactions between the fungus, plant, and soil water dynamics.

In the case of arbuscular mycorrhizal fungi (AMF), several studies have investigated the effect of AMFs on water potential: significantly higher leaf water potential was measured in snapdragon plants inoculated by AMF (El-Nashar, 2017). Boutasknit et al. (2020) studied the effect of AMF inoculation and water deficit on carob: leaf water potential was markedly affected by water conditions as well, but AMF-colonized carob plants showed more rapid recovery after removal of the water stress. The first experiment revealed that the growth benefit provided by living *Cadophora,* mainly under good water conditions, compared to autoclaved *Cadophora,* came with a cost manifested in decreased water potential under reduced water conditions. Based on this, inoculation with living *Cadophora* instead of autoclaved *Cadophora* is beneficial under good water conditions but has an extra cost and results in reduced water potential under water shortage. On the other hand, *Cadophora* seems to help the soil retain water. In the second experiment’s normal water treatment, all inoculations decreased water potential compared to the control. Hence, under an optimal water regime, all inoculations resulted in growth benefits and a water status cost. The inoculations did not decrease water potential in the reduced water treatment of the second experiment. Further, inoculation with the autoclaved DSE mix increased it. *Cadophora* had the strongest water potential decreasing effect when the water regime was not considered. Taken together, all inoculations resulted in growth benefits, with water status costs under optimal water supply but without water status costs under water shortage. Autoclaved DSE mix also appears to be a promising candidate for supporting plant development under water shortage since it not only promotes growth but also maintains water potential, likely due to its smaller effect on the shoot-to-root ratio. These results are particularly relevant for cabbage cultivation, but for a fine-scale understanding of the exact cost–benefit balance at different stages of development and under different environmental settings, further research is needed. Inoculation had no effect on soil moisture in Experiment I, but it had a significant interaction effect with water treatment in Experiment II. Under water shortage, living *Cadophora* increased, while under normal water supply, *P. macrospinosa* decreased soil moisture. We are not aware of any previous results highlighting such environment-dependent DSE effects. By affecting not only the host plants’ performance but also the quality of their environment, DSE may have more complex effects on ecological processes than previously expected.

## 5 Conclusion

We found that inoculations with different DSE species, their combination, and autoclaved DSE all had positive effects on cabbage development, mostly irrespective of water treatment. The effect mainly manifested in shoot development, resulting in an increased shoot-root ratio; however, positive effects were also observed on root development. DSE costs manifested as a decreased water status of the host plant, with the effect being most relevant under normal water regimes but not under water shortages. This is highly promising for agricultural use and potential management use in natural ecosystems. Living *Cadophora* and the autoclaved *Cadophora + P. macrospinosa* mix are the best candidates among the inoculations tested. The positive effects of *Cadophora* on shoot and root development, particularly under limited water conditions, may be attributed to enhanced nutrient uptake, improved water retention in the soil, or other mechanisms that warrant further exploration. Although *Cadophora* inoculation led to a decrease in plant water potential, this effect was primarily observed under normal water conditions, where the increased shoot-to-root ratio likely mitigated the negative impact. The costs associated with reduced water potential may be outweighed by the overall growth benefits, particularly under drought conditions. The autoclaved DSE mix was beneficial for plant growth overall, increasing both shoot and root development, and it increased water potential under water shortage. The two DSE fungi, similar to previous experiments, showed significant differences in their effects, which underpin the functional diversity of these common plant-associated fungi. Although the results are promising, they also highlight the need for optimizing any potential application of these fungi in agriculture. Given the promising results, these DSE inoculations could be explored as part of sustainable agricultural practices, particularly in drought-prone regions where water management is critical. However, further studies are needed to assess the cost-effectiveness and scalability of using these inoculants in commercial agriculture. Future research should prioritize large-scale screening efforts of diverse DSE species, including both living and autoclaved forms, across a wider range of host plants, particularly those important for agriculture. Additionally, studies should focus on varying environmental conditions such as soil type, nutrient availability, and plant developmental stages to better understand the dynamics of plant-fungi mutualistic symbiosis.

## Data Availability

The raw data supporting the conclusions of this article will be made available by the authors without undue reservation.
